# Acetylcholine Receptor Antibody Positivity, RNST Abnormality, and Thymoma Predict Generalization in Ocular Myasthenia Gravis: A South Korean Cohort Study

**DOI:** 10.3390/antib15040073

**Published:** 2026-08-11

**Authors:** Hyun Jin Shin, Chaerin Kwon, Jeeyoung Oh

**Affiliations:** 1 School of Medicine, Konkuk University, Chungju-si 27478, Republic of Korea; shineye@kuh.ac.kr (H.J.S.); 1224julia@naver.com (C.K.); 2 Department of Ophthalmology, Konkuk University Medical Center, Seoul 05030, Republic of Korea; 3 Research Institute of Medical Science, Konkuk University, Seoul 05029, Republic of Korea; 4 Department of Neurology, Konkuk University Medical Center, Seoul 05030, Republic of Korea

**Keywords:** ocular myasthenia gravis, generalized myasthenia gravis, acetylcholine receptor antibody, repetitive nerve stimulation test, thymoma

## Abstract

**Background:** Ocular myasthenia gravis (OMG) can remain restricted to ocular symptoms or progress to generalized myasthenia gravis (GMG). However, the rate of generalization and its predictors may vary across populations, and data on South Korean patients remain inadequate. This study investigated the conversion rate from OMG to GMG and identified clinical, serologic, electrophysiologic, and thymic predictors of this generalization in a South Korean cohort. **Methods:** This retrospective cohort study included 89 patients who initially presented with OMG and were followed up for at least 2 years. The primary outcome was conversion to GMG during follow-up. The investigated predictors included demographic variables, initial ocular manifestations, acetylcholine receptor antibody (AChR-Ab) status and titer, repetitive nerve stimulation test (RNST) findings, thymic abnormalities, and immunosuppressive treatment. Kaplan–Meier analyses and Cox proportional-hazards models were used to evaluate the GMG-free survival rate and risk factors for conversion. **Results:** During follow-up, 10 patients (11.2%) progressed to GMG, with this conversion occurring within 2 years in 8 patients (9.0%). All converters were AChR-Ab positive. AChR-Ab positivity, RNST positivity, thymic abnormality, and thymoma were associated with GMG conversion in univariable analyses. RNST positivity in any tested muscle (hazard ratio [HR] = 12.66, 95% confidence interval [CI] = 2.68–59.76, *p* = 0.001) and thymoma (HR = 10.67, 95% CI = 3.06–37.27, *p* < 0.001) was associated with generalization. These associations persisted in the restricted multivariable Cox model. **Conclusions:** The rate of GMG conversion was relatively low in this South Korean OMG cohort, and most conversions occurred within 2 years. The presence of AChR-Ab positivity, RNST abnormality, or thymoma may characterize high-risk patients who require closer monitoring during the early disease course.

## 1. Introduction

Myasthenia gravis (MG) is a B-cell-mediated autoimmune disease characterized by muscle weakness and fatigability, and is primarily caused by autoantibodies targeting the acetylcholine receptor (AChR) at the neuromuscular junction. Ocular MG (OMG) is a subtype of MG in which fatigability and weakness are restricted to the ocular muscles, and typically presents as fluctuating ptosis, diplopia, or both [1]. Although the manifestations remain ocular-only throughout the disease course in some patients, others progress to generalized MG (GMG), with the involvement of bulbar, respiratory, neck, axial, or limb muscles [2]. Because this generalization may require closer monitoring and more intensive systemic treatment, identifying patients at a high risk of conversion is clinically important [3].

The reported rate of conversion from OMG to GMG has varied widely across studies, ranging from approximately 20% to more than 60%, with most conversions occurring during the first 2 years after disease onset [4,5,6]. Several factors have been associated with generalization, including AChR-antibody (AChR-Ab) positivity, higher AChR-Ab titers, abnormal repetitive nerve stimulation test (RNST) findings, thymic abnormalities, thymoma, older age at onset, and immunosuppressive (IS) treatment [7,8,9]. The AChR-Ab status is a particularly relevant factor because it reflects the underlying autoimmune mechanism of MG and may help in stratifying the risk of systemic progression in patients who initially presented with ocular symptoms [10].

Geographic and racial differences might influence the clinical course of OMG. Findings from East Asian cohorts have suggested a lower rate of generalization and a greater tendency for OMG to remain restricted to ocular symptoms relative to Western cohorts [7,11,12]. However, data on the conversion rate and predictors of GMG progression in South Korean patients with OMG remain inadequate, particularly regarding the role played by the combination of AChR-Ab status, RNST positivity, and thymic pathology [13].

This study aimed to determine the rate and timing of conversion from OMG to GMG in a South Korean cohort with at least 2 years of follow-up. We also investigated clinical, serologic, electrophysiologic, thymic, and treatment-related factors associated with generalization, especially the AChR-Ab status and titer, RNST positivity, and thymoma. By characterizing these factors in an East Asian cohort, this study may facilitate individualized risk assessments and better follow-up strategies for patients who initially presented with OMG.

## 2. Materials and Methods

This retrospective cohort study was conducted at a single tertiary referral center: Konkuk University Medical Center in Seoul, South Korea. Medical records of patients diagnosed with MG and presenting with ocular symptoms between August 2005 and December 2023 were reviewed. The study was conducted in accordance with the principles of the Declaration of Helsinki and was approved by the Institutional Review Board of Konkuk University Medical Center. The requirement to obtain informed consent was waived due to the retrospective design of the study and the use of anonymized clinical data (Approval No. KUMC IRB 2025-03-011; Approval date 18 March 2025). The data used in this study were accessed for research purposes between 18 March 2025 and 18 June 2025.

### 2.1. Study Design and Patients

The included patients had initially presented with ocular manifestations compatible with OMG, such as fluctuating ptosis and/or diplopia, and had no clinical evidence of generalized myasthenic weakness. Patients were eligible for inclusion if they had a documented follow-up period of at least 2 years. Patients who developed GMG during follow-up were included as GMG converters. The exclusion criteria were as follows: (1) insufficient medical records at the time of diagnosis, (2) uncertain symptom classification at onset, (3) coexisting neurologic or ophthalmologic diseases that could interfere with an MG diagnosis, such as thyroid-associated ophthalmopathy, strabismus, chronic progressive external ophthalmoplegia, or cranial nerve palsy, or (4) drug-induced MG.

### 2.2. Diagnosis and Classification of MG

MG was diagnosed based on characteristic fluctuating and fatigable muscle weakness, particularly involving the ocular muscles, together with available serological, electrophysiological, and supportive clinical evidence. In patients without AChR-Ab positivity or abnormal RNST findings, the diagnosis of OMG was based on a comprehensive assessment of characteristic fluctuating or fatigable ptosis and/or diplopia, exclusion of alternative causes, ice-pack test findings, and documented clinical improvement following pyridostigmine treatment. Pyridostigmine responsiveness was considered supportive evidence rather than a stand-alone confirmatory criterion. All diagnoses were finalized by consensus between a neuro-ophthalmologist (H.J.S.) and a neurologist (J.Y.O.) [14,15]. OMG was defined as myasthenic involvement restricted to ocular manifestations, including ptosis, diplopia, or orbicularis oculi weakness at the initial presentation, while GMG conversion was defined as the development of generalized myasthenic symptoms beyond ocular involvement during follow-up, including bulbar symptoms, respiratory symptoms, neck weakness, limb weakness, or other generalized muscle weakness.

The final disease status was classified using the Myasthenia Gravis Foundation of America (MGFA) clinical classification system [2]: MGFA class I was considered OMG and MGFA class II or higher was considered GMG. Patients who initially presented with OMG but later developed MGFA class II or higher disease during follow-up were classified as GMG converters, whereas those who remained at MGFA class I throughout the follow-up period were classified as nonconverters. MGFA classes II, III, and IV represent mild, moderate, and severe generalized weakness, respectively, and are further subdivided into “a” for predominant limb or axial muscle involvement and “b” for predominant bulbar or respiratory involvement. MGFA class V denotes the most severe form of GMG, which is characterized by profound generalized weakness requiring intubation.

### 2.3. Clinical and Laboratory Assessments

Baseline and follow-up data were collected from medical records, including demographic characteristics, ages at symptom onset and diagnosis, duration of follow-up, initial ocular manifestations, AChR-Ab status and titer, RNST findings, associated autoimmune or thyroid disease, thymic abnormalities, thymectomy, and IS treatment before GMG conversion or censoring. AChR-Ab positivity was defined according to the institutional laboratory reference range, with titers ≥ 0.5 nmol/L considered positive. The RNST was applied to the orbicularis oculi, trapezius, and abductor digiti minimi muscles. RNST positivity was defined as a CMAP decrement of >10% in any tested muscle, and the muscle showing the greatest decrement was recorded [15]. For one patient who underwent RNST at an outside institution, only a qualitative result was available; this result was included in the binary RNST analysis but excluded from the quantitative decrement analysis. Serum AChR-Ab levels and RNST were measured at the initial visit as part of the diagnostic evaluation and before the initiation of corticosteroids or nonsteroidal immunosuppressive therapy at our institution. Thymic abnormalities were assessed primarily using contrast-enhanced chest computed tomography and categorized as absent, thymic hyperplasia, or thymoma. If thymectomy had been performed, radiologic findings were confirmed by a pathologic diagnosis when this was available. Single-fiber EMG (SFEMG) was not routinely performed during the study period and was therefore not included in the analysis.

### 2.4. Outcome Measurements

The primary outcome was conversion from OMG to GMG during follow-up. The time to GMG conversion was defined as the interval from the onset of ocular symptoms or the initial OMG presentation to the first documentation of generalized symptoms. Patients without GMG conversion were censored at the last follow-up. Secondary outcomes included the overall conversion rate, 2-year conversion rate, time to conversion, and GMG-free survival rate. Potential predictors included demographic variables, age at onset, initial ocular manifestations, AChR-Ab status and titer, RNST positivity, thymic abnormality, thymoma, autoimmune or thyroid comorbidity, and IS treatment before conversion or censoring.

### 2.5. Statistical Analyses

Continuous variables are presented as mean ± standard deviation or median and interquartile range (IQR), and categorical variables are presented as numbers and percentages. Between-group comparisons were performed using the Mann–Whitney U test for continuous variables and Fisher’s exact test or the Fisher–Freeman–Halton exact test for categorical variables, as appropriate. GMG-free survival was estimated using the Kaplan–Meier method, and patients without GMG conversion were censored at the last follow-up. Survival curves were compared using the log-rank test. Risk factors for GMG conversion were assessed using Cox proportional-hazards regression models, with the results presented as hazard ratios (HRs) and 95% confidence intervals (CIs). The proportional-hazards assumption was assessed for individual covariates and the overall model using Schoenfeld residual-based tests with rank-transformed event times. Because only a small number of conversion events occurred, multivariable Cox regression was performed using restricted models that included only clinically relevant variables showing significant associations in univariable analyses. AChR-Ab positivity was excluded from the main multivariable Cox model because all patients who converted to GMG were AChR-Ab positive, resulting in complete separation. A two-sided *p* value < 0.05 was considered statistically significant. Statistical analyses were performed using SPSS software (version 27.0; IBM Corporation, Armonk, NY, USA).

## 3. Results

### 3.1. Baseline Characteristics

The analyses included 89 patients with OMG (Table 1). The cohort comprised 43 males (48.3%) and 46 females (51.7%), with a mean age of 51.2 ± 17.2 years at diagnosis and 50.1 ± 17.4 years at symptom onset. Thirty-nine patients (43.8%) had early-onset OMG (age < 50 years), whereas the remaining 50 patients (56.2%) had late-onset OMG (age ≥ 50 years). The median interval from symptom onset to treatment was 2.0 months (IQR, 0.8–8.5 months), and the median total follow-up duration was 69.0 months (IQR, 30.0–120.0 months). At presentation, ptosis was observed in 81 patients (91.0%), diplopia in 63 (70.8%), and both ptosis and diplopia in 55 (61.8%). Among the 89 patients, 36 (40.4%) were AChR-Ab positive and 24 (27.0%) had abnormal RNST findings. Seventeen patients (19.1%) had both findings, 19 (21.3%) were AChR-Ab positive with normal RNST findings, and 7 (7.9%) had abnormal RNST findings despite AChR-Ab negativity. The remaining 46 patients (51.7%) were negative for both AChR-Ab and RNST and fulfilled the prespecified clinical diagnostic criteria. Thymic abnormalities were identified in 21 patients (23.6%). During follow-up, 10 patients (11.2%) progressed to GMG, including 8 (9.0% of the entire cohort) who converted within 2 years. The median time to GMG conversion among converters was 4.0 months (IQR, 2.0–9.2 months).

### 3.2. Comparing GMG Converters and Nonconverters

The results from comparisons between GMG converters and nonconverters are summarized in Table 2. There was no significant difference between the two groups in sex distribution, age at diagnosis, age at symptom onset, early- versus late-onset status, symptom-to-treatment interval, total follow-up duration, or initial ocular symptom pattern. In contrast, AChR-Ab positivity was significantly more common in GMG converters than in nonconverters (100.0% vs. 32.9%, *p* < 0.001). RNST positivity was also more common among GMG converters, both for any tested muscle (80.0% vs. 20.3%, *p* < 0.001) and for orbicularis oculi stimulation (80.0% vs. 16.5%, *p* < 0.001). Thymic abnormalities were more common in GMG converters (70.0% vs. 17.7%, *p* = 0.001), particularly thymoma (50.0% vs. 6.3%, *p* < 0.001 for thymic-abnormality subtype distribution). AChR-Ab titers among seropositive patients were numerically higher in converters but the difference did not reach statistical significance. Overall steroid and nonsteroidal immunosuppressant use did not differ significantly between the groups.

### 3.3. Risk Factors for GMG Conversion

Univariable analyses showed that AChR-Ab positivity, RNST positivity, thymic abnormality, and thymoma were associated with conversion from OMG to GMG (Table 3). The HR for AChR-Ab positivity was not estimable because all patients who progressed to GMG were seropositive, although the log-rank test was significant (*p* < 0.001). RNST positivity in any tested muscle (HR, 12.66; 95% CI, 2.68–59.76; *p* = 0.001), thymic abnormality (HR, 8.59; 95% CI, 2.22–33.29; *p* = 0.002), and thymoma (HR, 10.67; 95% CI, 3.06–37.27; *p* < 0.001) were significant predictors, whereas demographic factors, the initial ocular symptom pattern, and AChR-Ab titer among seropositive patients were not. In a restricted multivariable Cox proportional-hazards model, RNST positivity in any tested muscle (adjusted HR, 13.62; 95% CI, 2.69–68.89; *p* = 0.002) and thymoma (adjusted HR, 12.02; 95% CI, 2.91–49.59; *p* < 0.001) remained independently associated with conversion to GMG. Schoenfeld residual-based tests showed no evidence of violation of the proportional-hazards assumption for RNST positivity (*p* = 0.916), thymoma (*p* = 0.727), or the multivariable model as a whole (global test, *p* = 0.940). AChR-Ab positivity was not included in the multivariable model because the absence of conversion events among seronegative patients resulted in monotone likelihood, precluding reliable estimation of its HR.

### 3.4. GMG-Free Survival Rates and Risk-Factor-Based Kaplan–Meier Analyses

**Figure 1:**
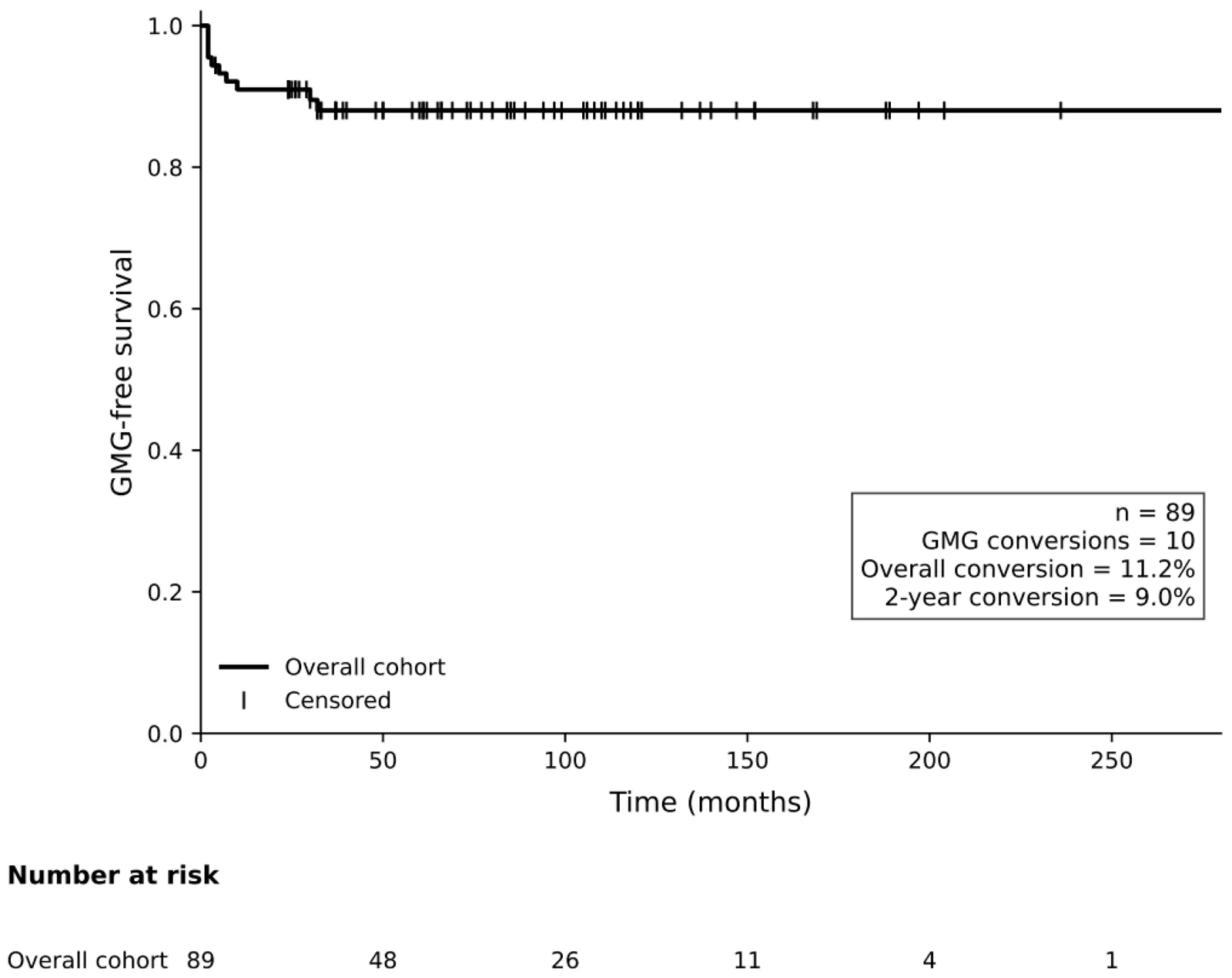
Kaplan–Meier curve for generalized myasthenia gravis (GMG)-free survival rate in the overall ocular myasthenia gravis (OMG) cohort. Among 89 patients with OMG, 10 progressed to GMG during follow-up, corresponding to an overall conversion rate of 11.2%. Eight patients converted within 2 years, yielding a 2-year conversion rate of 9.0%.

**Figure 2:**
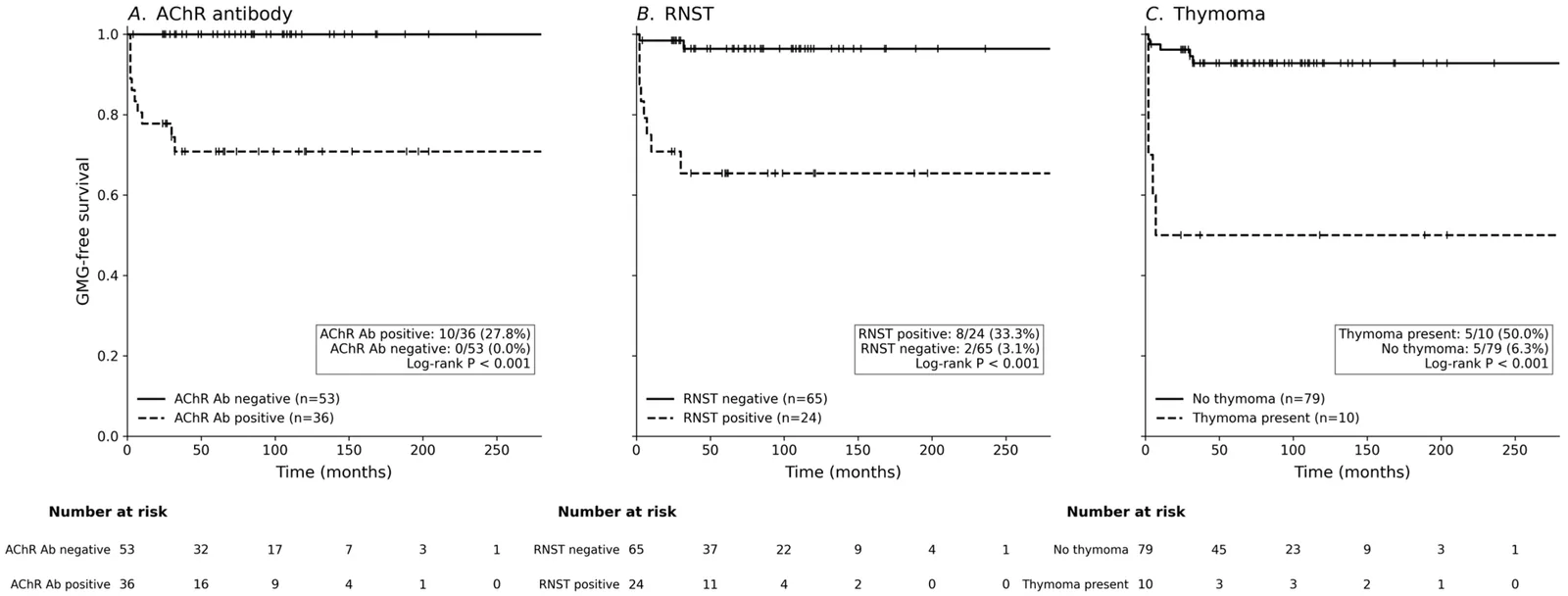
Kaplan–Meier curves for GMG-free survival rate according to major risk factors. (**A**) GMG-free survival rate according to acetylcholine receptor antibody (AChR-Ab) status. The GMG-free survival rate was significantly lower in AChR-Ab-positive than in AChR-Ab-negative patients (log-rank *p* < 0.001). (**B**) GMG-free survival rate according to repetitive nerve stimulation test (RNST) findings. GMG-free survival rate was significantly lower in RNST-positive than in RNST-negative patients (log-rank *p* < 0.001), with RNST positivity defined as positivity in any tested muscle. (**C**) GMG-free survival rate according to thymoma status. The GMG-free survival rate was significantly lower in patients with than without thymoma (log-rank *p* < 0.001).

Kaplan–Meier analyses of the overall OMG cohort showed that the GMG-free survival rate remained high during follow-up, with 10 of 89 patients progressing to GMG and 8 patients converting within 2 years (Figure 1). The findings of risk-factor-based Kaplan–Meier analyses are shown in Figure 2. The GMG-free survival rate was significantly lower in AChR-Ab-positive than in AChR-Ab-negative patients (log-rank *p* < 0.001) (Figure 2A). It was especially notable that all patients who progressed to GMG were AChR-Ab positive. Similarly, the GMG-free survival rate was significantly lower in RNST-positive than in RNST-negative patients (log-rank *p* < 0.001) (Figure 2B). Thymoma was also strongly associated with a lower probability of remaining restricted to ocular manifestations, since patients with thymoma demonstrated a significantly reduced GMG-free survival rate compared to those without thymoma (log-rank *p* < 0.001) (Figure 2C).

## 4. Discussion

This study aimed to determine the rate and predictors of generalization in South Korean patients who initially presented with OMG. Ten (11.2%) of 89 patients progressed to GMG, with this conversion occurring within 2 years in 8 patients (9.0%). AChR-Ab positivity, RNST positivity, thymic abnormality, and thymoma were associated with GMG conversion, while demographic factors, initial ocular symptom pattern, and AChR-Ab titer among seropositive patients were not significant predictors. In a restricted multivariable analysis, RNST positivity and thymoma remained independently associated with generalization, suggesting that serologic, electrophysiologic, and thymic markers can help to identify high-risk OMG patients despite the overall low conversion rate in this South Korean cohort.

The association between AChR-Ab positivity and GMG conversion suggests that seropositivity reflects a broader potential for systemic autoimmune disease, even when the initial presentation is clinically restricted to ocular symptoms [8,12,16,17]. All of the patients in our cohort who progressed to GMG were AChR-Ab positive. However, the AChR-Ab titer among seropositive patients was not significantly associated with conversion, suggesting that the presence of AChR-Ab is more informative than a single quantitative titer measurement for predicting generalization [3,17,18]. This may be partly explained by how disease duration and timing of AChR-Ab measurement can influence AChR-Ab levels [3,12]. RNST positivity also showed an association with conversion, supporting the notion that subclinical neuromuscular junction dysfunction already extends beyond clinically apparent ocular involvement in some patients with OMG [7,19]. This interpretation is consistent with previous findings, including Guo et al. reporting abnormal RNS findings, AChR-Ab seropositivity, thymoma, and adult-onset OMG as risk factors for generalization [8].

Thymoma was the strongest thymus-related predictor in our analysis [20,21]. From an immunopathologic perspective, thymoma may contribute to systemic progression via impaired immune tolerance, defective selection of AChR-specific autoreactive T cells, increased autoantibody production, and broader activation of the immune system [22]. These mechanisms may explain why patients with thymoma are more likely to progress from ocular-restricted disease to GMG [11]. Our findings are also consistent with AChR-Ab positivity, positive RNS findings, and thymoma being identified as major predictors of OMG generalization in a Singaporean Asian cohort [7]. Together, these results suggest that the AChR-Ab status, electrophysiologic abnormalities, and thymic pathology should be considered together when estimating the risk of generalization in patients who initially presented with OMG [3,7].

Several factors that were previously proposed as predictors of generalization were not significantly associated with GMG conversion in our cohort. We found that age at onset and a late-onset status (age ≥ 50 years) were not significant predictors of GMG conversion, which contrasts with previous Western and Chinese studies identifying older age as a risk factor [5,8,9,12,21]. This discrepancy may reflect the predominantly adult-onset profile of our cohort, with an age at diagnosis of 51.2 ± 17.2 years, as well as the small number of conversion events reducing the ability to detect age-related effects [3,7]. Sex was not associated with GMG conversion in our cohort, although female sex has previously been proposed as a risk factor [12,16,23], which could be related to the immunomodulatory effects of female hormones [12,24]. However, the evidence regarding sex as a predictor of generalization remains inconsistent, since several studies have failed to demonstrate a statistically significant association [3,5,7,22]. The initial ocular symptom pattern, including combined ptosis and diplopia, did not predict GMG conversion in our cohort. Although combined ocular symptoms have been reported more frequently among converters, this association often disappears after multivariable adjustment [8,21,24]. However, this remains an area of debate, with Feng et al. reporting that bilateral ptosis at onset was associated with a 2.05-fold higher risk of generalization, suggesting that bilateral ocular involvement reflects more severe autoimmune activity [5].

A similar discrepancy was observed for the AChR-Ab titer. Although AChR-Ab positivity was strongly associated with GMG conversion in our study, the AChR-Ab titer among seropositive patients was not a significant predictor. This contrasts with some previous reports of titers being higher in patients who converted. For example, Teo et al. found that the mean AChR-Ab titer was higher in converters than in nonconverters (15.51 vs. 9.38 nmol/L), with each 1-nmol/L increase associated with a modest increase in the risk of generalization (HR = 1.027, *p* = 0.002) [7]. A Thai study also suggested using a high titer threshold of 8.11 nmol/L as a predictor of conversion [25]. These discrepancies may reflect differences in assay methods, including radioimmunoassays rather than more sensitive cell-based assays [26,27], as well as variations in the disease duration and timing of AChR-Ab measurement [5].

The conversion rates in the present South Korean cohort were 9.0% at 2 years and 11.2% overall, broadly similar to the corresponding rates of 7.7% and 10.6% reported in a Singaporean cohort [7]. Although these findings are consistent with previous reports of lower generalization rates in some Asian cohorts than in Western cohorts, cross-study comparisons should be interpreted cautiously because of differences in follow-up duration, treatment strategies, inclusion criteria, and AChR-Ab prevalence (Table 4). Therefore, the observed variation cannot be attributed solely to ethnicity or geographic region. Nevertheless, several biological and environmental factors may contribute to regional variation in generalization risk. Immunogenetic background may be relevant, as Japanese studies have associated HLA-DRw9 with persistent ocular manifestations and HLA-DRw8 with generalization [28]. Differences in serologic profiles may also contribute, because AChR-Ab positivity has generally been reported less frequently in Asian than in Western OMG cohorts [5,7,18,26]. Given the association between AChR-Ab positivity and generalization [10], variation in seropositivity could partly account for the differences observed across cohorts. Environmental factors, including latitude, ultraviolet-light exposure, and vitamin D status, have also been proposed as potential contributors to geographic variation in the risk of secondary generalization [29].

Our findings support a risk-stratified approach to the management of patients who initially presented with OMG. Patients without AChR-Ab positivity, RNST abnormality, or thymoma may be managed with routine monitoring and education regarding the symptoms of generalization. In contrast, patients with high-risk features such as AChR-Ab positivity, RNST abnormality, or thymoma may require closer observation, particularly during the first 2 years after onset when most conversions occur. Careful assessments for subtle bulbar or limb weakness are especially important in this subgroup. The RNST may serve not only as a diagnostic test but also as a risk-stratification tool, since RNST positivity may indicate subclinical neuromuscular junction involvement beyond the clinically apparent ocular disease.

This risk-stratified framework also has implications for treatment decisions. Previous studies have suggested that immunosuppressive therapy can reduce the risk of generalization in patients with OMG. For example, Kupersmith et al. reported that the 2-year incidence of GMG was lower in prednisone-treated patients than in untreated patients [4], and Mee et al. similarly concluded that receiving immunotherapy for OMG reduced the rate of conversion to GMG [31]. A more recent systematic review and meta-analysis also supported the potential benefit of immunosuppressive therapy in preventing progression from OMG to GMG [32]. Although treatment decisions should be individualized, these findings suggest that early systemic immunotherapy should be considered in selected high-risk OMG patients [33].

Treatment-related confounding should also be considered. Most patients received corticosteroids, and nearly half received additional nonsteroidal immunosuppressants, which may have reduced the observed risk of generalization. Although treatment exposure did not differ significantly between converters and nonconverters, this finding should not be interpreted as evidence that immunosuppressive therapy had no effect on generalization. Treatment initiation, timing, duration, dose, and subsequent modification were determined according to clinical circumstances and may have varied with disease severity and treatment response, resulting in confounding by indication and time-dependent treatment changes. Consequently, the observed associations of RNST positivity and thymoma with GMG conversion cannot be fully separated from the potential effects of early immunotherapy and may reflect both intrinsic disease risks and treatment-related factors. Given the small number of generalization events and the retrospective design, treatment effects could not be reliably adjusted for in the Cox model. Prospective studies incorporating standardized treatment protocols, detailed longitudinal treatment exposure, and time-dependent analyses are needed to determine whether early or sustained immunosuppressive therapy modifies the risk of GMG conversion and to clarify the independent prognostic value of RNST positivity and thymoma.

This study was limited by its retrospective, single-center design and the requirement for at least 2 years of follow-up, which may have introduced selection bias and led to an underestimation of the GMG conversion rate if early dropouts converted or sought care elsewhere. Retrospective documentation may also have missed subtle or transient nonocular weakness, with both factors potentially contributing to an underestimation of the GMG conversion rate. Additionally, while the cohort encompassed a wide age range, most patients had adult-onset disease, and the low event count limited statistical power to evaluate age and sex as independent predictors.

Statistical and methodological constraints also affected our analyses. With only 10 generalization events and two covariates, the final Cox model had approximately five events per variable, leading to wide confidence intervals and potential model instability; thus, independent associations should be interpreted cautiously. Furthermore, because all converters were AChR-Ab positive, their effect could not be reliably estimated via conventional Cox regression due to monotone likelihood. Although methods such as Firth penalized Cox regression can address complete separation, the small event count precluded robust multivariable estimation. Confounding by indication and the impact of time-varying treatment exposure or thymectomy could also not be evaluated. Finally, SFEMG—which is more sensitive than RNST for detecting neuromuscular transmission defects in OMG—was not routinely performed, potentially underdetecting abnormalities in RNST-normal patients; likewise, MuSK and LRP4 antibodies were not systematically tested in AChR-Ab-negative patients, leaving some seronegative cases incompletely characterized serologically. Future multicenter prospective studies with larger cohorts, comprehensive diagnostic panels (SFEMG, MuSK/LRP4), and standardized treatment data are essential to confirm the independent prognostic value of AChR positivity, abnormal RNST, and thymoma, and to determine if early therapeutic intervention or heightened surveillance reduces generalization risk in these high-risk patients.

## 5. Conclusions

The rate of OMG generalization was low in this South Korean cohort, with most conversions occurring during the first 2 years. AChR-Ab positivity, RNST abnormality, and thymoma were associated with GMG conversion, while RNST abnormality and thymoma retained statistically significant associations with generalization in a restricted multivariable Cox proportional-hazards model. These findings may help inform risk-stratified follow-up and individualized treatment decisions for patients with OMG, particularly those with serologic, electrophysiologic, or thymic risk factors.

## Figures and Tables

**Table 1:** Baseline characteristics of the entire ocular myasthenia gravis (OMG) cohort.

Characteristic	Value (n = 89)
Sex	43 males (48.3%), 46 females (51.7%)
Age at diagnosis, years	51.2 ± 17.2, 53.0 [38.0–64.0]
Age at symptom onset, years	50.1 ± 17.4, 52.0 [38.0–61.0]
Onset age group	Early onset in 39 (43.8%), late onset in 50 (56.2%)
Symptom-to-treatment interval, months	2.0 [0.8–8.5]
Total follow-up duration, months	69.0 [30.0–120.0]
Initial ocular symptom pattern	Ptosis only in 26 (29.2%), diplopia only in 8 (9.0%), both in 55 (61.8%)
Diplopia pattern at presentation	None in 26 (29.2%), horizontal in 17 (19.1%), vertical in 20 (22.5%), both in 26 (29.2%)
AChR-Ab status and titer	Positive in 36 (40.4%) with a titer of 7.3 [2.1–10.6] nmol/L
Thymic abnormality	Any abnormality in 21 (23.6%), thymic hyperplasia in 11 (12.4%), thymoma in 10 (11.2%)
RNST positivity	Any muscle in 24 (27.0%), orbicularis oculi in 21 (23.6%), trapezius in 12 (13.5%), abductor digiti minimi in 5 (5.6%)
IS treatment during ocular phase	Steroid in 76 (85.4%), nonsteroidal agent in 41 (46.1%)
Nonsteroidal immunosuppressant	Azathioprine in 26 (29.2%), mycophenolate mofetil in 13 (14.6%), tacrolimus in 20 (22.5%), cyclosporine in 3 (3.4%)

Note: Data are n (%), mean ± standard deviation, or median [IQR] values, as appropriate. AChR-Ab positivity was defined as a titer ≥ 0.5 nmol/L. AChR-Ab, acetylcholine receptor antibody; IQR, interquartile range; RNST, repetitive nerve stimulation test; IS, immunosuppressive.

**Table 2:** Comparing generalized myasthenia gravis (GMG) converters and nonconverters.

Variable	GMG Converters (n = 10)	GMG Nonconverters (n = 79)	*p*
Sex, male	3 (30.0)	40 (50.6)	0.318
Age at diagnosis, years	46.0 [36.2–52.0]	54.0 [39.0–65.0]	0.170
Age at symptom onset, years	44.0 [36.2–52.0]	53.0 [38.5–62.5]	0.203
Early onset (age < 50 years)	6 (60.0)	33 (41.8)	0.324
Symptom-to-treatment interval, months	3.7 [1.5–5.5]	2.0 [0.7–9.2]	0.721
Initial symptom: ptosis only/diplopia only/both	2 (20.0)/1 (10.0)/7 (70.0)	24 (30.4)/7 (8.9)/48 (60.8)	0.881
AChR-Ab positive	10 (100.0)	26 (32.9)	<0.001
AChR-Ab titer in seropositive patients, nmol/L	8.6 [6.5–11.2]	6.4 [1.8–9.6]	0.185
RNST positivity	8 (80.0)	16 (20.3)	<0.001
Thymic abnormality	7 (70.0)	14 (17.7)	0.001
Thymic-abnormality subtype: absent/hyperplasia/thymoma	3 (30.0)/2 (20.0)/5 (50.0)	65 (82.3)/9 (11.4)/5 (6.3)	<0.001
Thymectomy	7 (70.0)	8 (10.1)	<0.001
Steroid treatment	8 (80.0)	68 (86.1)	0.636
Nonsteroidal immunosuppressant	7 (70.0)	34 (43.0)	0.177

Note: Data are n (%) or median [IQR] values. *p* values were calculated using the Mann–Whitney U test for continuous variables, Fisher’s exact test for 2 × 2 categorical variables, and the Fisher-Freeman-Halton exact test for multicategory categorical variables.

**Table 3:** Results from univariable Cox proportional-hazards analyses of risk factors for conversion from OMG to GMG.

Variable	Events/Total	Value	*p*
Sex, male	4/43	0.45 (0.12–1.74)	0.246
Age at onset, per 10-year increase	10/89	0.83 (0.58–1.18)	0.297
Late onset (age ≥ 50 years)	4/50	0.52 (0.15–1.82)	0.304
Both ptosis and diplopia at presentation	7/55	1.41 (0.36–5.46)	0.619
AChR-Ab positivity	10/36	NE	<0.001 ^†^
AChR-Ab titer, per 1-nmol/L increase	10/36	1.13 (0.97–1.32)	0.126
RNST positivity	8/24	12.66 (2.68–59.76)	0.001
Thymic hyperplasia	2/11	1.72 (0.37–8.12)	0.491
Thymoma	5/10	10.67 (3.06–37.27)	<0.001

Note: Data are n/n values or hazard ratios (95% confidence intervals) from analyses of univariable Cox proportional-hazards models. For binary variables, the reference groups were female sex, age < 50 years, absence of both ptosis and diplopia, AChR-Ab negativity, RNST negativity, absence of thymic abnormality, absence of thymic hyperplasia, absence of thymoma, and no prior immunotherapy. ^†^, HR for AChR-Ab positivity was not estimable (NE) because all GMG converters were AChR-Ab positive; the *p* value was obtained using the log-rank test.

**Table 4:** Reported generalization rates in selected Western and East Asian OMG cohorts.

Region	Study: First Author (Year)	Country	Sample Size (N)	Follow-Up Duration	Conversion Rate	Key Characteristics
**Western**	Kupersmith (2003) [4]	USA	94	≥2 years	18–36%	IS therapy significantly reduced conversion
	Nagia (2015) [3]	USA	158	60.5 m	20.9%	Lower rate in a recent cohort compared with historical data
	Mazzoli (2018) [12]	Italy	168	15 years	18.4%	Late onset (age ≥ 50 years) and AChR-Ab positivity associated with conversion
	Sommer (1997) [30]	Germany	78	8.4 years	31%	Most (84.7%) generalizations occurred within 2 years
	Díaz-Maroto (2023) [23]	Spain	62	4.5 years	50%	High conversion rate in a Mediterranean cohort
**East Asian**	Hong (2008) [11]	South Korea	202	11.8 m	11.4–20%	East Asian tendency to remain restricted to ocular manifestations
	Teo (2018) [7]	Singapore	155	40.8 m	10.6%	7.7% at 2-year follow-up; low conversion rate
	Ding (2020) [9]	China	223	4 years	17.0%	Patients received IS therapy
	Guo (2022) [8]	China	572	14.5 m	25.2%	Multicenter study; adult onset had higher risk
	Kemchoknatee (2025) [24]	Thailand	155	5 years	20.6%	AChR-Ab positivity was the main risk factor

Note: m, months.

## Data Availability

The minimal anonymized dataset required to reproduce the findings of our study has been deposited in a public repository (Figshare) and is accessible via the following DOI: http://doi.org/10.6084/m9.figshare.32573280.

## Abbreviations

The following abbreviations are used in this manuscript: AChR, acetylcholine receptor; Ab, antibody; ADM, abductor digiti minimi; CI, confidence interval; GMG, generalized myasthenia gravis; HR, hazard ratio; IQR, interquartile range; MG, myasthenia gravis; MGFA, Myasthenia Gravis Foundation of America; OMG, ocular myasthenia gravis; RNST, repetitive nerve stimulation test.
